# Novel Sensor-Enabled *Ex Vivo* Bioreactor: A New Approach towards Physiological Parameters and Porcine Artery Viability

**DOI:** 10.1155/2015/958170

**Published:** 2015-11-01

**Authors:** Raghavendra Mundargi, Divya Venkataraman, Saranya Kumar, Vishal Mogal, Raphael Ortiz, Joachim Loo, Subbu Venkatraman, Terry Steele

**Affiliations:** School of Materials Science and Engineering, Nanyang Technological University, Singapore 639798

## Abstract

The aim of the present work is to design and construct an *ex vivo* bioreactor system to assess the real time viability of vascular tissue. Porcine carotid artery as a model tissue was used in the *ex vivo* bioreactor setup to monitor its viability under physiological conditions such as oxygen, pressure, temperature, and flow. The real time tissue viability was evaluated by monitoring tissue metabolism through a fluorescent indicator “*resorufin.*” Our *ex vivo* bioreactor allows real time monitoring of tissue responses along with physiological conditions. These *ex vivo* parameters were vital in determining the tissue viability in sensor-enabled bioreactor and our initial investigations suggest that, porcine tissue viability is considerably affected by high shear forces and low oxygen levels. Histological evaluations with hematoxylin and eosin and Masson's trichrome staining show intact endothelium with fresh porcine tissue whereas tissues after incubation in *ex vivo* bioreactor studies indicate denuded endothelium supporting the viability results from real time measurements. Hence, this novel viability sensor-enabled *ex vivo* bioreactor acts as model to mimic *in vivo* system and record vascular responses to biopharmaceutical molecules and biomedical devices.

## 1. Introduction

In the last couple of years, biomedical research is directed towards recitation of cells with biomaterials for the therapeutic advancement and regeneration of tissues [1, 2]. This area of research has promoted interaction among scientific and industrial communities leading to innovation of new biomaterials in healthcare [1–5]. Various* in vivo* models have been developed to understand vascular biology and effect of therapeutic agents delivered by biomedical devices [6–8]; among these models,* ex vivo* tissue culture system has been described as an alternative model to* in vivo* animal and* in vitro* cell culture models to evaluate vascular responses in relation to biomechanical and biochemical factors [7, 8].

The* ex vivo* bioreactor simulates physiological environment to support testing of tissues and support structures and organs* in vitro*. The* ex vivo* model uses arterial tissues and allows evaluation of cellular interactions in three-dimensional architecture of tissue; these biological responses can be performed in controlled physiologic conditions, namely, oxygen, pressure, temperature, and flow. Yamawaki et al. [9] and Wright et al. [10] reported applications of* ex vivo* tissue culture models to study the proliferative changes in vascular smooth muscle cells (SMC) as an important phenomenon in atherosclerosis and neointimal formation in response to the culture media.

The* ex vivo* tissue culture model has also been used to study physiological mechanisms of vascular tissue [11, 12] and these studies significantly support material-tissue interactions and the role of vascular endothelial layer in mediating vasodilation [13]. Yazdani and Berry [14] investigated the effect of stent design on SMC proliferation in* ex vivo* porcine carotid arteries using carotid self-expanding stents. The* ex vivo* bioreactor with excised tissues can be used to evaluate and predict clinical outcomes of the biomedical devices. Such* ex vivo* bioreactors maintain physiological conditions to study product prototypes for better performance in preclinical and clinical studies by minimizing product development cycle; also this step aids in reducing preclinical costs by implementing different prototype modifications in* ex vivo* studies. The existing vascular bioreactors lack monitoring real time tissue viability in presence of physiological conditions; to address this challenge, we designed a simple* ex vivo* bioreactor set up with biosensors alignment to monitor real time viability along with physiological conditions. To our knowledge, the* ex vivo* tissue culture model has not been used to evaluate real time tissue viability along with physiological parameters in the sensor-enabled bioreactor. The objective of the present work is to design and construct a sensor-enabled* ex vivo* bioreactor to monitor real time vascular tissue responses; our* ex vivo* perfusion setup provides a new and cost-effective approach compared with current* in vivo* animal model by maintaining the physiological parameters oxygen, pressure, temperature, and flow for various applications, that is, cold storage solutions for organ transplants [15] or assessing mitochondrial activity of the neonatal arteries [16] and toxicity [17].

## 2. Experimental Section

### 2.1. Materials

Masterflex peristaltic pump (EW-77202-50) along with silicon, Tygon tubings (numbers 16, 25) was purchased from Cole-Parmer (IL, USA). Electronics components consisted of breadboard with DAQ: NI6052E was obtained from National Instruments (Singapore). Biosensor components, isolated dissolved oxygen sensor, and pressure sensor with reusable transducer (RT 2000) were obtained, respectively, from World Precision Instruments (FL, USA) and Argon Medical Devices (Singapore). The thermistor probe (SP4042) used in the bioreactor assembly was obtained from Thermometrics (CA, USA) and high glucose Dulbecco's modified eagle medium (DMEM) was purchased from Invitrogen (CA, USA). Hypotubes used in the bioreactor assembly were custom-made of blunt stainless steel 12 G needle (inner diameter 2.16 mm) to hold swine carotid arteries.

### 2.2. Tissue Harvesting and Handling

Porcine carotid arteries were harvested from 80 ± 10 kg, 6-month-old swine of either sex from Primary Industries Pte Ltd. (Singapore). Carotid arteries were collected with length varying from 8 to 12 cm from the animals free from any ailment. Arteries without any branches were selected and rinsed with sterile solution of phosphate buffer saline (pH 7.4) and 2% antibiotic-antimycotic (ABAM, penicillin-streptomycin; Invitrogen, California). Excess fat was removed from excised porcine arteries by using scissors and cleaned arteries were placed in the sterile medium containing Dulbecco's modified eagle medium (DMEM, Invitrogen), 10% fetal bovine serum, and 2% ABAM.

**Figure 1:**
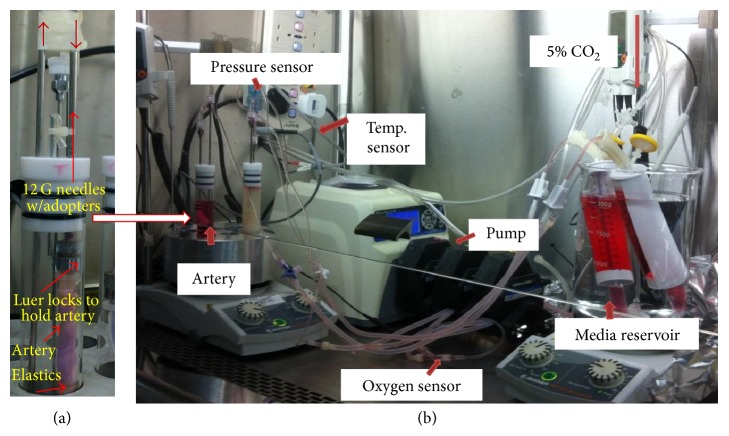
Sensor-enabled* ex vivo* bioreactor setup. (a) Artery fixed in the bioreactor tube supporting the Luer locks; selected artery with desired dimensions is connected to hypotubes and firmly attached using elastics to avoid leakages. (b) Bioreactor overall setup with sensor attachment; hypotube containing artery is placed in an aluminum block maintained on temperature controlled heating mantle. The inlet and outlets of the hypotubes are connected to the media reservoir through peristaltic pump to facilitate circulation of media.

The cleaned arteries were placed in a fresh sterile Teflon plate and rinsed with sterile PBS and 2% ABAM several times by flushing inside and surrounding the artery. Further, each cleaned artery was placed in separate tube containing media at 4°C for overnight. The same procedure was repeated to clean multiple arteries. The cleaned artery with approximate length of 8–10 cm was selected and connected to the upper hypotube and tied using elastics as shown in Figure 1(a). The artery connected to the hypotube was placed inside the sterile glass chamber containing media (DMEM, 10% FBS, and 2% ABAM) and transferred to aluminum cage set at 37°C. Further, the bioreactor glass chamber tube was connected to media reservoir and peristaltic pump using pressure tubings to circulate media in the bioreactor setup. The media circulated through the perfusion system and whole setup was observed for intact circulation without any leaks.

### 2.3. Bioreactor Setup and Sensor Attachment

The* ex vivo* bioreactor setup with porcine artery was assembled in the sterile biohood and media reservoir was placed in the glass beaker containing preheated (37°C) sterile purified water. Overall setup of our* ex vivo* bioreactor is displayed in Figure 1(b) indicating each part. In the bioreactor, media inlet was connected to the hypotube port and outlet was diverted to top of the reservoir using pressure tubing. 5% CO_2_ incubator line was connected to the reservoir through 0.22 *μ*m sterile filter. Pressure sensor and in-line temperature sensors were aligned to the* ex vivo* setup and oxygen sensor along with flow meter was connected in line with fluid flow through the peristaltic pump. Oxygen was monitored by the OXELP oxygen electrode and ISO2 dissolved oxygen meter and for pressure and temperature sensing RT 2000 reusable transducer and injectate sensor cable SP4042 were used. Indirect flow measurement was carried out by Masterflex peristaltic pump. Signals were conditioned by an in-house assembled printed circuit board (PCB) (electronic components: Element 14, Singapore; PCB: Seed Technology Inc., China) and computer acquisition was performed by the NI PCI-6052E DAQ (National Instruments).

Media with specific composition (150 mL DMEM, 10% FBS, and 2% ABAM) were circulated to the reservoir by using peristaltic pump. The bioreactor glass chamber containing artery was connected to the media reservoir and gradually pump was adjusted to generate a flow rate of 40 mL/min. The culture medium used in the bioreactor was replaced for every 24 h and the medium was assessed for any bacterial contamination at the end of every perfusion step. The bioreactor tube was observed for any leakage for 10 minutes and resazurin solution was transferred to the circulating media by using 1 mL sterile syringe. All the operations involving resazurin addition, circulation were done in sterile conditions.

### 2.4. Real Time Tissue Viability Assessment

**Figure 2:**
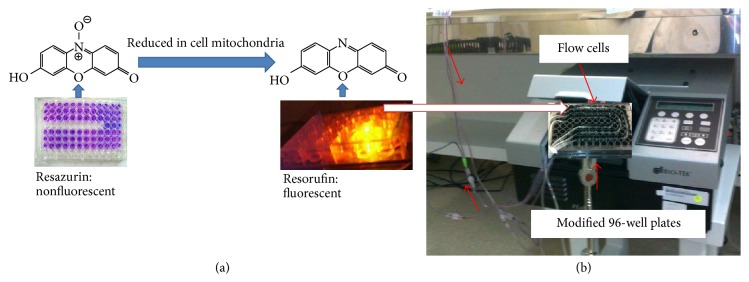
Real time tissue viability detection. (a) Resazurin converting to resorufin by cell metabolic activity. (b) Flow cells and fluorimeter setup for real time measurements of florescent resorufin by flow cells.

Resazurin is reduced to generate highly fluorescent resorufin and this irreversible reaction of resazurin to resorufin is proportional to aerobic respiration in the bioreactor system. The long-wavelength spectral properties of resorufin and high sensitivity of the assay result in little interference from coloured components in the bioreactor samples. We have used resazurin to resorufin conversion as indicator of tissue viability; in addition, resorufin in solution is not quenched by oxygen or chloride and is unaffected by changes in media concentration. The schematic representation of conversion of resazurin to resorufin by the cell metabolic activity along with flow cell setup is depicted in Figure 2(a). The fluorescent resorufin formed in the circulating media was measured by using fluorimeter set up in Figure 2(b) at specific excitation and emission wavelengths (*λ*_excitation_-540 nm; *λ*_emission_-590 nm). In the present bioreactor setup, the flow cells along with tubings were designed to achieve the inline connection with the bioreactor system to observe real time porcine tissue viability. In the current bioreactor setup, sterile filtered resazurin (50 *μ*M) was used as cell viability indicator and real time measurements were carried out every five minutes by measuring fluorescence of the medium using flow cell reader and slope values were computed at specified time intervals.

### 2.5. Biosensor Circuits and MATLAB Programming

**Figure 3:**
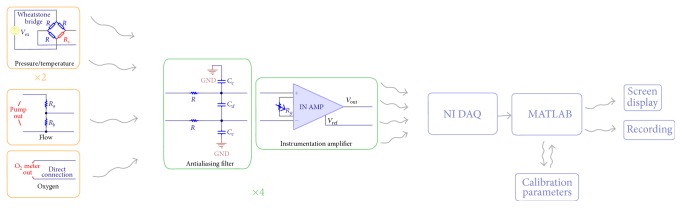
Schematic view of sensor signal assemblage and processing in the* ex vivo* bioreactor.

Although sensors dedicated to each parameter are commercially available, our approach offers a more homogenous, easily extensible, and cost-efficient solution. The schematic view of sensor signal assemblage and signal processing is shown in Figure 3. The first stage of the conditioning circuit is specific to each sensor and remains limited to a Wheatstone bridge (4 resistors). In our current biosensor setup, a differential scheme is altered to accommodate a wide range of sensors and the analog input signal goes through antialiasing filters. These signals were amplified and buffered by an instrumentation amplifier (INA116) with an adjustable gain and resulting signals were compatible with the DAQ card inputs in terms of input range, bandwidth to convert acquired signals into digital domain (see Supplementary Figures S1 and S2 in Supplementary Material available online at http://dx.doi.org/10.1155/2015/958170). Finally, raw signals were processed in our MATLAB bioscope. Signal noise was reduced by digital filtering process and conversion from voltage to defined physical quantity was performed and the bioscope allows instant signal monitoring, synchronized recording, and assisted sensor calibration.

### 2.6. Histological* Ex Vivo* Tissue Evaluation

For histological assessment, the ring shaped porcine carotid artery tissues before the experiment and after the viability run were harvested and fixed in 4% paraformaldehyde for paraffin processing. Tissue sections were used for routine hematoxylin and eosin (H&E) and Masson's trichrome (MT) staining (IMCB, Biopolis, Singapore). Two slides per tissue specimen were prepared and all the slides were screened and analyzed for the histological findings.

## 3. Results and Discussion

### 3.1. Bioreactor Design and Capabilities

The* ex vivo* bioreactors function as model to* in vivo* conditions exerted in vascular* in vivo* conditions by simulating physiological conditions such as oxygen, pressure, temperature, and flow. These bioreactors significantly influence in recording critical vascular responses in* ex vivo* conditions in response to biomedical devices such as stents and drug eluting balloons intended for intraluminal delivery. Hence, vascular tissue viability in such* ex vivo* bioreactor setup is vital in evaluating the performance of these devices. Biomedical devices are evaluated for their performance in terms of tissue viability, vascular responses, drug release, pharmacokinetics, and tissue penetration [18, 19]. In this work, we have demonstrated the design and model of sensor-enabled* ex vivo* bioreactor system to record real time tissue viability with reference to physiological parameters.

The biosensors were connected in-line with the circulation media to record physiological parameters along with real time tissue viability. The physiological parameters in the* ex vivo* perfusion system were optimized to mimic the* in vivo* vascular conditions such as oxygen, pressure, temperature, and flow. The pulsatile flow was created with peristaltic pump by operating at a speed of 25 rpm and oxygen supply from incubator to the reservoir was attained by using air pump. In* ex vivo* bioreactor setup hemodynamic environment with a mean flow of 40 mL/min, internal pressure of 120/70 mmHg, and temperature of 37 ± 0.5°C was maintained. The MATLAB interface was designed to account for mean values of physiological parameters with respect to time during the progress of experiment. The pressure wave forms were optimized by varying flow rate and size of the pressure tubing to achieve appropriate physiological conditions [11]. The wave forms generated in the bioreactor were able to exhibit 100 beats per minute. However, the physiological conditions reported in swine carotid artery are blood flow (284 mL/minute), carotid artery pressure (CAP, 101 mmHg), internal pressure (120/80 mmHg) (see Supplementary Figure S3), heart rate (116 beats per minute), and shear stress (26 to 69 dynes/cm^2^). Hence, in the current* ex vivo* sensor-enabled bioreactor, we have achieved physiological conditions related to CAP, heart rate, and shear stress. In view of high* in vivo* flow of 284 mL/min it was difficult to set up similar flow in* ex vivo* conditions due to media leakages [20–22].

### 3.2. Factors Affecting* Ex Vivo* Tissue Viability

**Figure 4:**
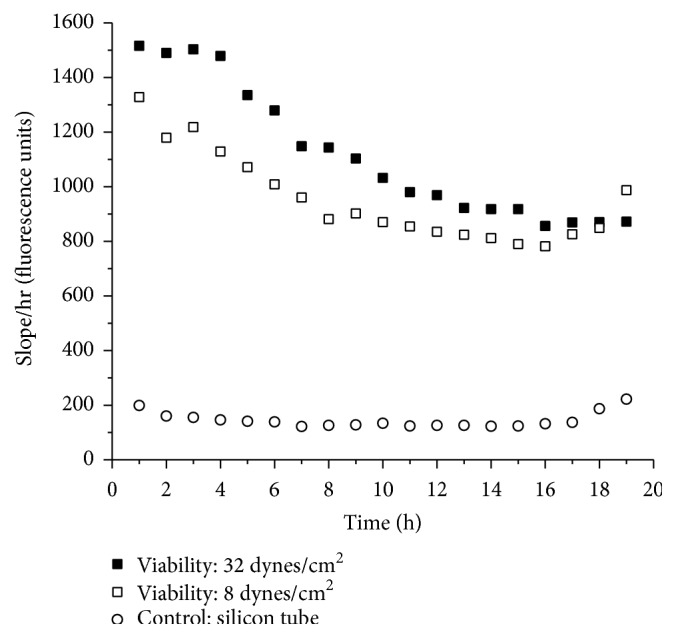
Representative graph showing effect of shear stress on tissue viability. Real time tissue viability monitored after tissues stored for 24 h (32 dynes/cm^2^) and 48 h (8 dynes/cm^2^) to demonstrate* ex vivo* bioreactor functionality to record variations in tissue responses at given conditions.

To study the real time tissue response, the harvested artery was fixed to the bioreactor and circulating media from bioreactor were aligned to flow via flow cells situated in fluorimeter. The florescence intensity of resorufin as cell viability indicator was measured at specific time intervals (Figure 2(a)). In the present bioreactor system resazurin (50 *μ*M) converts to fluorescent resorufin in the cell mitochondria; the real time fluorescent values recorded during* ex vivo* perfusion were computed to slope values for control (silicon tube) and tissue.  Figure 4 depicts the tissue response at different shear stress in the* ex vivo* perfusion system and approximate vascular wall shear stress at artery wall was determined by using Hagen-Poiseuille approximation by computing flow rate, vessel diameter, and viscosity of the medium [21]. The real time response from the tissue at shear stress of 8 dynes/cm^2^ and 32 dynes/cm^2^ indicates effect of shear stress induced by the perfusion system. In case of control (silicon tube) the real time measurements show constant fluorescent values due to absence of any metabolic activity. In case of relatively higher vascular shear stress (32 dynes/cm^2^) the tissue viability dropped higher compared to low vascular stress of 8 dynes/cm^2^ and this could lead to denudation of endothelium [23]. Thus, initiation of SMC death as well as luminal and abluminal progression of cell death could suggest the damage to the endothelium initiating chain of reactions leading to cell death [16]. Hence, the physiological parameters along with shear stress, tissue harvest time are vital in deciding the rate of tissue metabolism and viability in* ex vivo* perfusion system [19].

**Figure 5:**
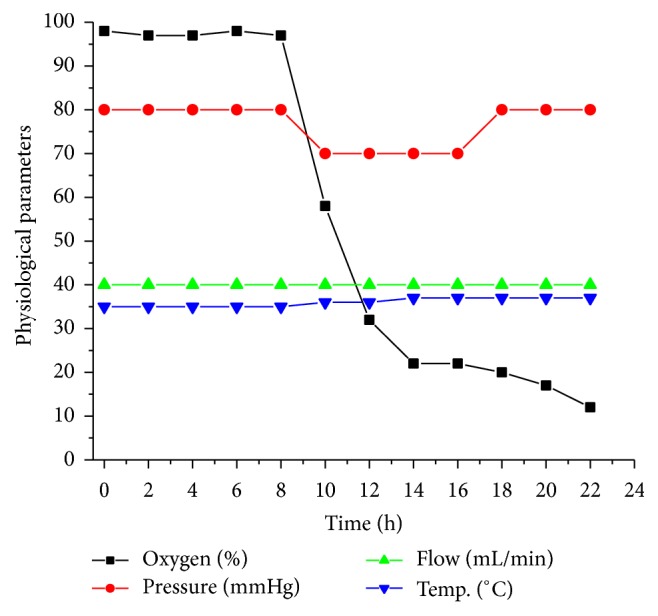
Representative graph showing physiological parameters observed in the* ex vivo* bioreactor during real time swine tissue viability measurements. All the data points are average of specified data points as set in Bioscope with MATLAB program and average values are reported for every-2-hour intervals.

The physiological parameters observed in the* ex vivo* bioreactor are displayed in Figure 5 and indicate that the oxygen levels in bioreactor consistently decrease to 20% over 20 h time interval. The diastolic pressure was 90 ± 10 mmHg, flow is consistent at 40 mL/min, and temperature was 37 ± 0.5°C.

**Figure 6:**
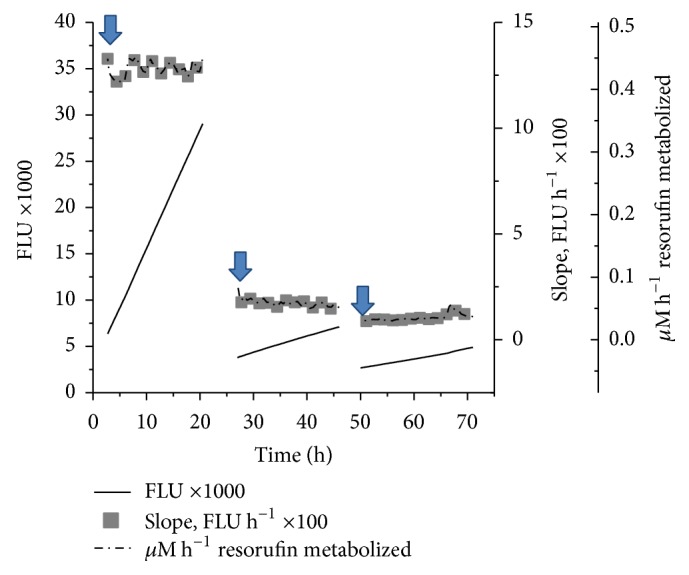
Representative real time tissue responses and resorufin metabolism in the* ex vivo* bioreactor. The rate of metabolic activity is given by the slope and metabolic activity per unit time is correlated to the time duration of viability experiment in* ex vivo* conditions similar to those displayed in Figure 5. The media change at each interval is indicated in the figure with arrow mark.

To further investigate factors affecting the tissue viability, cell viability indicator resorufin metabolized was analyzed and presented in *μ*M/h (Figure 6). At initial time points, higher resorufin (0.45 *μ*M/h) was metabolized indicating higher tissue viability, but with reduced oxygen levels lower resorufin was metabolized resulting in low tissue viability. In the current* ex vivo* bioreactor setup, we observed a clear trend as a result of metabolically active cells reducing nonfluorescent resazurin to pink fluorescent resorufin in the presence of mitochondrial dehydrogenase enzymes and over the time interval the metabolic activity is reduced (see Supplementary Figure S4).

**Figure 7:**
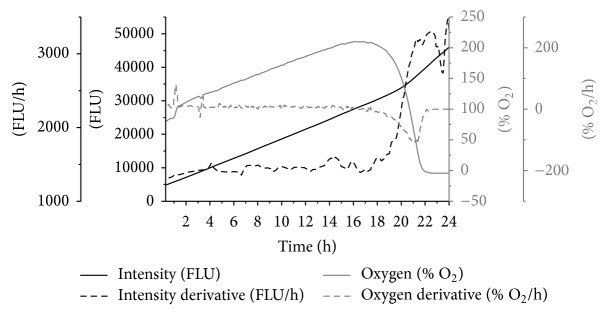
Representative tissue viability with oxygen supply in the bioreactor.

Figure 7 illustrates the effect of oxygen on rate of metabolic activity of the porcine artery in* ex vivo* conditions. As the rate of dissolved oxygen in the perfusing media increases, there was an increase in fluorescent intensity; the depletion in oxygen levels was observed at 18 h due to possible microbial contamination following which there was an exponential increase in the fluorescence. The oxygen and fluorescence derivate have consistent values until 18 h of* ex vivo* tissue perfusion with real time viability measurements and thereafter both of the derivatives shift the trend.

**Figure 8:**
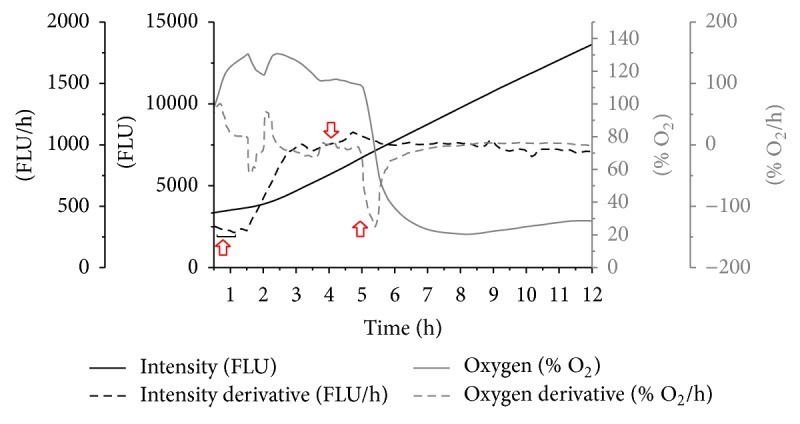
Representative graph showing effect of oxygen, nitrogen on resazurin metabolism and tissue viability. Initial 1 h silicon tube was used as control to indicate no metabolic activity; after 1 hr swine artery was connected to* ex vivo* bioreactor under oxygen supply to monitor tissue metabolic activity until 4 hr. After 4 h oxygen supply was terminated and nitrogen is supplied to observe tissue responses in absence of oxygen.

These results imply the vital role of oxygen in maintaining the tissue metabolism to maximum extent; the tissue metabolism in presence and absence of oxygen with baseline values is presented in Figure 8. Initially for 1 h the baseline fluorescence was recorded with silicon tube in place of artery as control to indicate absence of any metabolic activity in the bioreactor. After 1 h, the swine artery was connected and real time fluorescence intensity was monitored; the values increased drastically due to tissue metabolic activity in presence of physiological parameters. Further, the oxygen supply is terminated and nitrogen inflow was maintained in the bioreactor to observe tissue response; there was sudden decrease in the fluorescent intensity as a result of lack of any tissue metabolism.

Our experimental finding clearly shows the effect of oxygen on the viability of the porcine tissue. The interpretations of the present tissue viability study in sensor-enabled bioreactor indicate the feasibility of real time monitoring of vascular tissue responses with physiological conditions. The technique used to assess the real time tissue viability by measurement of fluorescence of the cell viability indicator is important tool to predict the tissue responses in sensor-enabled bioreactor.

### 3.3. Histological Studies

**Figure 9:**
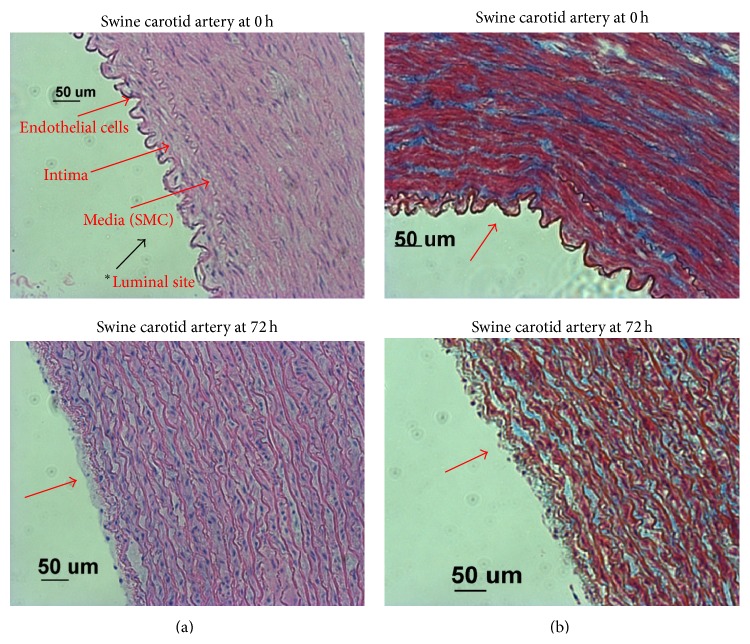
Representative histology slides of* ex vivo* swine carotid artery tissue in bioreactor at 0 h and at 72 h. (a) Hematoxylin and eosin staining. Intact endothelium at 0 h and denuded endothelium at 72 h, causing a lower tissue viability with increase in artery diameter from 2.62 to 4.52 mm. (b) Masson's trichrome staining; cytoplasm is indicated with red colour, blue colour indicates collagen, and dark brown spots are nuclei.

Figure 9 shows the fresh artery stained with H&E and MT and all the images are facing the lumen side. In case of H&E staining intact endothelium is observed with nuclei (blue colour). In case of MT stain, dark brown spots and blue lining, respectively, indicate the cell nuclei and collagen, and the light red colour specifies cytoplasm. The vascular endothelium is clear without any disruption indicating the intact endothelial layer (intima); the lumen diameter of arteries was measured in histological slides and found to be increased in the perfusion system from 2.62 mm to 4.52 mm due to continuous shear stress induced by the media flow. Tissue section of the artery was exposed to DMEM up to 72 h in the bioreactor and histological studies clearly indicate the disruption of endothelial layer. This denudation of endothelial layer could be due to injury caused by the consistent flow and shear stress exerted by the perfusion system and dimension changes of the artery in the* ex vivo* bioreactor. The* ex vivo* viability is consistently decreasing to 30% at the end of 72 h; the decreasing oxygen levels in the bioreactor also aid the decreased viability trend. The* ex vivo* bioreactor simulates the physiological conditions and helps to maintain the tissue viability, but as consequences of inherent tissue sensitivity to hypoxia the endothelium is denuded [16].

## 4. Conclusions

The present work demonstrates the design and function of* ex vivo* bioreactor system capable of maintaining physiological conditions along with hemodynamic forces to mimic the* in vivo* vascular conditions. We have designed the sensors and programmed with MATLAB for real time account of physiological parameters in the* ex vivo* bioreactor. Tissue metabolism and real time viability were studied for the first time with the capability of recording vascular responses in the* ex vivo* setup. Initial experiments with porcine carotid artery indicate that shear stress and oxygen levels in the bioreactor were vital parameters in deciding the tissue viability. The histological findings in this study support the real time tissue viability measurements by sensor-enabled bioreactor. Such systems can play a major role in recording vascular responses in screening biopharmaceutical drugs and biomedical devices.

## Supplementary Material

The information on sensor connection, data acquisition, ex-vivo bioreactor generated pressure wave forms and schematic on resazurin conversion to resorufin are all included in Supplementary Material.
