# Supplementary material for: Novel Sensor-Enabled Ex Vivo Bioreactor: A New Approach towards Physiological Parameters and Porcine Artery Viability
Source: Biomed Res Int. 2015 Nov 1;2015:958170. doi: 10.1155/2015/958170 (PMC4644552; doi:10.1155/2015/958170)
Supplement: Supplementary file 1 — The information on sensor connection, data acquisition, ex-vivo bioreactor generated pressure wave forms and schematic on resazurin conversion to resorufin are all included in Supplementary Material. [file 958170.f1.pdf]

1. # Appendix

2. **Novel Sensor-Enabled *Ex-Vivo* Bioreactor-A New Approach towards Physiological Parameters and Porcine Artery Viability**

3. Raghavendra C. Mundargi, Divya Venkataraman, Saranya Kumar, Vishal Mogal, Raphael Ortiz, Joachim Loo, Subbu Venkatraman and, Terry Steele

4.

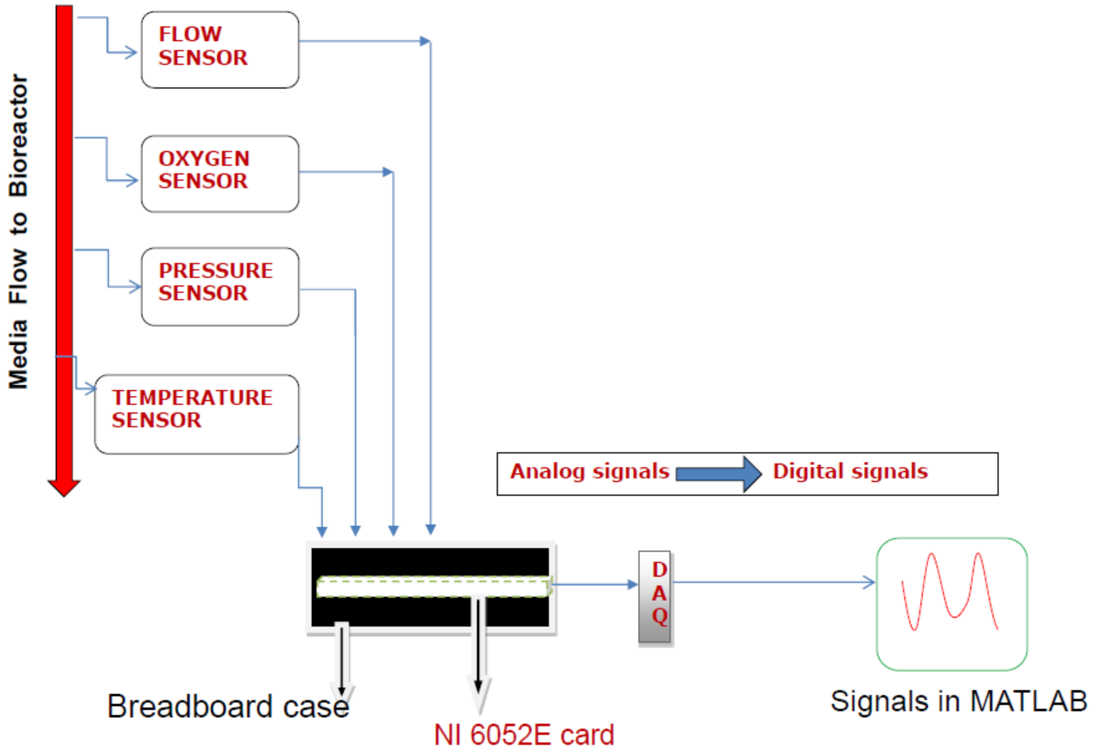

Figure S1: Flow diagram for sensors connection to data acquisition card (DAQ).

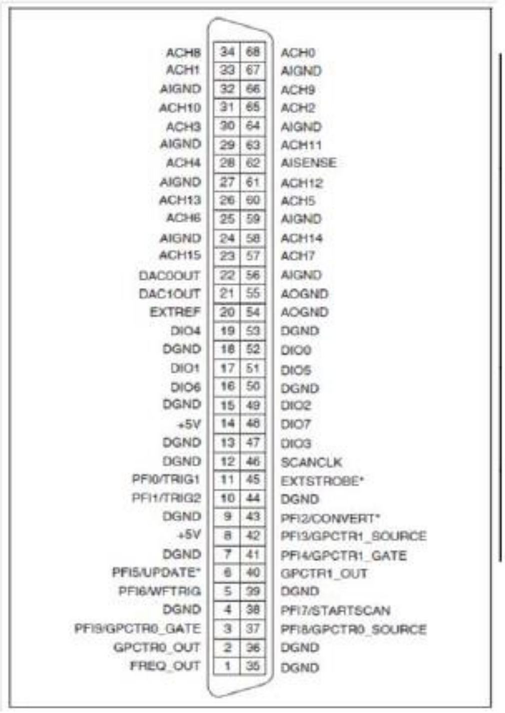

Sensors connections to NI6052E	
1	Oxygen<br>ACHO ----68<br>ACH8 ----34
2	Pressure<br>ACH1 ----33<br>ACH9 ----66<br>Excitation:<br>DAC0OUT --22<br>AOGND ----55
3	Temperature<br>ACH2 ----65<br>ACH10---31
4	Flow<br>ACH3----30<br>ACH11---63

Figure S2: I/O connector pin assignment and sensors connections to the NI6052E card.

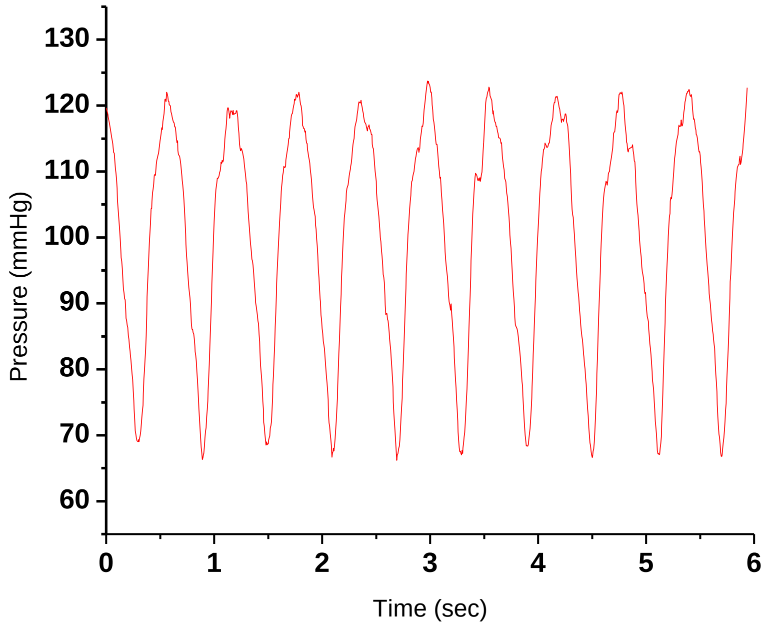

Figure S3: Pressure wave forms generated in the sensor-enabled online *ex-vivo* bioreactor.

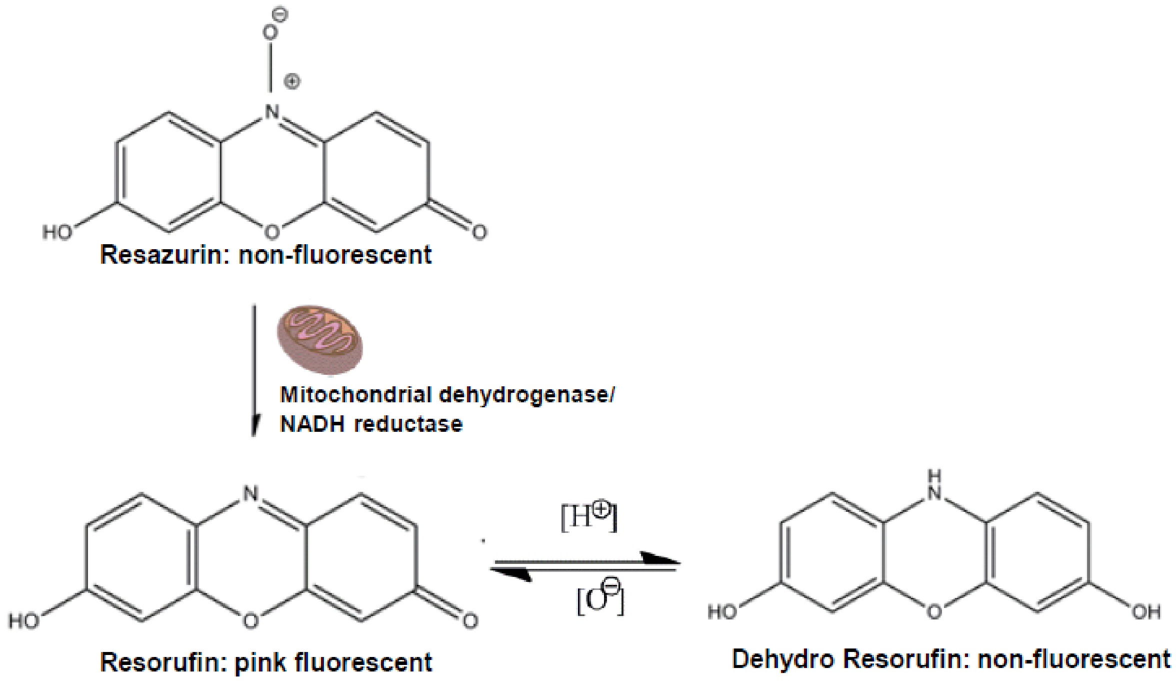

5. **Figure S4**: Schematic showing conversion of resazurin to fluorescent resorufin.
